# Neuronal correlates of intensification and acceptance of symptoms during exposure therapy in patients with obsessive-compulsive disorder

**DOI:** 10.3389/fpsyg.2024.1256046

**Published:** 2024-02-05

**Authors:** Susanne Karch, Maximilian Maywald, Caroline Schwartz, Clara Heil, Jakob Neumüller, Daniel Keeser, Sarah Garcia, Nadja Tschentscher, Oliver Pogarell, Marco Paolini, Ulrich Voderholzer

**Affiliations:** ^1^Department of Psychiatry and Psychotherapy, University Hospital LMU, Munich, Germany; ^2^Schoen Clinic Roseneck, Prien am Chiemsee, Germany; ^3^Department of Radiology, University Hospital LMU, Munich, Germany

**Keywords:** obsessive-compulsive disorder, fMRI, neuroscience, acceptance strategy, exposure therapy

## Abstract

**Introduction:**

Cognitive behaviour therapy with exposure and response prevention is efficient in treating patients with obsessive-compulsive disorder (OCD). Nevertheless, it would be helpful for many patients to complement the therapeutic treatment with acceptance strategies to further increase the therapeutic benefit. The aim of the present study was to examine neurobiological responses to acceptance and intensification strategies during symptom provocation alongside the psychotherapeutic process.

**Method:**

A total of 23 patients diagnosed with OCD (subtype: washing/contamination fear) was instructed to utilise either an acceptance strategy (ACS) or an intensification strategy (INS) to cope with their emotional and cognitive reactions to personalised symptom-triggering and neutral pictures. Fourteen patients participated twice: at the beginning [T1] and at the end [T2] of an inpatient multimodal treatment including cognitive behaviour therapy with response prevention to assess functional variations.

**Results:**

For the contrast of T1 and T2, ACS showed increased brain activity in the left inferior frontal gyrus (IFG), left caudate body, and posterior cingulate gyrus (PCC). They also showed decreased activity in the left anterior insula. INS showed decreased activation in right lingual gyrus and right caudate body. At T2, ACS showed increased activation compared to INS in the left cerebrum: IFG, caudate nucleus, middle and superior temporal gyrus, and PCC/cuneus. For the comparison of T1 and T2, the ACS revealed increased brain activity in the left IFG, left caudate body, and right inferior parietal lobe. It showed decreased activity in the left anterior insula. The INS revealed decreased activity in right lingual gyrus and right caudate body.

The psychometric questionnaires suggested that patients were able to reduce obsession, compulsion, and depression symptoms. Furthermore, patients rated the ACS as more useful for themselves compared with the INS.

**Conclusion:**

The increased left IFG activity using ACS (T1 vs. T2) could be interpreted as a better inhibitory top-down process, while the increased PCC response might be due to a better reappraisal strategy after therapy. ACS seems to mobilise neuronal activations under therapy, especially in the left hemisphere. Both strategies showed reductions in emotional networks as a neuronal correlate of therapy success. Overall, ACS may be more efficient than INS, as rated by the patients and as in accordance with neurobiological findings.

## 1 Introduction

Obsessive-compulsive disorder (OCD) is characterised by the presence of uncontrollable, reoccurring thoughts (*obsessions*) and/or behaviours (*compulsions*), e.g., washing, checking, or mental rituals in order to reduce distress (American Psychiatric Association, 2013). For patients, compulsive behaviours may seem to serve as a neutralising tool for reducing the perceived distress of experiencing daily aversive and obsessive thoughts in the first place (Goodman et al., 2014). However, these behavioural acts are neither adaptive coping strategies, nor are they inherently pleasurable and may not even produce the desired counterbalancing effect (Goodman et al., 2014). In line with the refined metacognitive model of Wells (1997, 2000), individuals give a lot of meaning to their intrusive thoughts and equate thoughts with future actions, which triggers the anxiety-reducing compulsive behaviours (Fisher and Wells, 2005a,b). This process maintains OCD symptomatology and is maladaptive (Fisher and Wells, 2005a,b).

### 1.1 Cognitive behaviour therapy and its neurobiological underpinnings

Cognitive behaviour therapy (CBT) with exposure and response prevention has been recommended as psychotherapeutic treatment of choice for patients with OCD. Within the CBT approach, various strategies can be used including “Exposure and Response Prevention” (ERP), requiring patients to undergo systematic symptom provocation through exposure to relevant stimuli to learn to resist the urge to perform the compulsions. It has been assumed that the main effect for this therapeutic intervention is habituation to provoked negative emotions and reduced responses (Foa and Kozak, 1986). In addition, cognitive therapy for OCD aims to identify and reduce dysfunctional appraisals of intrusive thoughts, and the impact of compulsive behaviour. Studies show that both ERP and cognitive therapy are effective interventions in the treatment of OCD (Rosa-Alcazar et al., 2008; Brennan et al., 2014; McKay et al., 2015; Ost et al., 2015). Overall, CBT and ERP may cause significant symptom improvements in OCD patients and are efficient in treating OCD. Nevertheless, a relatively high number of patients does not show a substantial benefit from any psychotherapy (Schwartz et al., 2013; Fineberg et al., 2015; Key et al., 2017).

A recent meta-analysis on neuronal activity during *symptom provocation* showed higher activation in the right caudate body/putamen/insula, and lower activation in the left orbitofrontal cortex (OFC), left inferior frontal gyrus (IFG), left caudate body/middle cingulate cortex (MCC), right middle temporal gyrus (MTG), middle occipital gyrus (MOG), and right lateral occipital gyrus (LOG) in OCD patients compared to healthy controls (Yu et al., 2022). Overall, the authors concluded that OCD patients showed elevated dorsal striatal activation during symptom provocation. By contrast, the washing subgroup revealed higher activation in frontal, temporal and posterior cortical structures, as well as in right caudate body compared to healthy subjects (Yu et al., 2022).

Kwon et al. (2009) reported dysfunctions regarding the OFC, anterior cingulate cortex (ACC), thalamus, dorsolateral prefrontal cortex (DLPFC), caudate nucleus, as well as in parietal regions both during resting state and symptom provocation in patients with OCD as compared to healthy controls. This may indicate an imbalance between dorsal and ventral fronto-striatal circuits (Kwon et al., 2009).

Aouizerate et al. (2004) examined how the OCD-related abnormalities in brain activity can be interpreted. According to their assumptions the OFC might be particularly attributed to the consequences of action, thereby sub-serving decision-making. By contrast, the ACC is particularly relevant in situations with conflicting options and a high probability of making an error. Processing of relevant information has been related to the DLPFC among other areas. The caudate nucleus is helpful for the integration of cortical information and the control of behaviour (Aouizerate et al., 2004).

A meta-analysis by Frank et al. (2014), focusing on emotion regulation in healthy individuals, revealed signal change in the amygdala/parahippocampal gyrus that was specifically influenced by the regulation strategy (down- vs. up-regulation). By contrast, cortical regions (e.g., superior frontal gyrus, cingulate, and premotor areas) exhibited enhanced activity regardless whether a down- or upregulation was intended (Frank et al., 2014). Overall, decreased activity in limbic areas during an acceptance strategy is supposed to account for the efficacy of regulation (e.g., Frank et al., 2014; Morawetz et al., 2017).

### 1.2 Mindfulness-based cognitive therapies and their neurobiological underpinnings

Mindfulness-based cognitive therapies (MBCT) teach people to experience and accept thoughts and feelings regarding their symptoms without judgement, which might lead to reduced anxiety and performance of compulsive acts (Fairfax, 2008; Twohig et al., 2010). Key et al. (2017) demonstrated that MBCT facilitate the disengaging from cognitive routines and the acceptance of internal experiences. These skills may be valuable in treating OCD patients, as individuals describe getting “stuck” in repetitive thoughts and rituals (Key et al., 2017). The acceptance of negative emotions can probably improve/simplify inhibitory learning processes. Studies that examined the effect of acceptance and commitment therapy showed that acceptance-based interventions can lead to positive effects in patients with OCD (Twohig et al., 2010).

Kulz et al. (2019) reported that patients with OCD and residual symptoms after CBT, which were randomised to either an MBCT group or to a psychoeducational group (OCD-PE), reported higher self-rated improvements and higher quality of life in the MBCT group, as compared to a psychoeducational group post treatment (Kulz et al., 2019).

A recent meta-analysis examining neurobiological underpinnings of emotional acceptance included the data of 422 subjects in 13 experiments (Messina et al., 2021). Participants were instructed to take on a non-judgmental attitude towards on-going emotional experiences as an emotion regulation strategy. The aim was to clarify the involvement of executive areas and of the default mode network (DMN) in the acceptance strategy (Messina et al., 2021). Their results indicated that the use of acceptancy strategies did not reveal increased neuronal responses in brain areas associated with executive processes when compared to control conditions (e.g., focus on emotion). However, executive areas were affected during acceptance when specifically compared to neutral reactions (e.g., react naturally) (Messina et al., 2021).

By contrast, the comparison of neurobiological responses during the acceptance strategy as compared to various control conditions (e.g., strategy to react naturally or focus on emotions) demonstrated decreased responses in the posterior cingulate cortex (PCC)/precuneus during acceptance strategy. Overall, the authors concluded that these results indicate that higher-level executive cortical processes are not a distinctive feature of acceptance, whereas functional deactivations in the PCC/precuneus constitute its specific neural substrate (Messina et al., 2021). This may suggest a complementary role of limbic portion of the default system during the modification of emotional states (Messina et al., 2021).

### 1.3 Neurobiological responses change after psychotherapy

Different studies examined the neuronal changes following CBT through symptom provocation in participants with OCD. Their results generally supported the hypothesis of normalised activation in various brain regions including bilateral ACC, left OFC, bilateral DLPFC, right insula, bilateral nucleus accumbens, and left supramarginal gyrus (SMG) after therapy as compared to before (Baioui et al., 2013; Schiepek et al., 2013; Morgieve et al., 2014).

A systematic review on neural substrates of successful therapy showed a post-treatment decrease of symptoms and enhanced responses in the ventral circuits during symptom provocation, as well as mainly increased activity in dorsal circuits during cognitive processing (Thorsen et al., 2015). These effects seemed to be common in both psychotherapy and pharmacological approaches (Thorsen et al., 2015). The authors concluded that the results of their review indicate that dysfunctions in neural function and structure are partly reversible and state-dependent for affective symptoms (Thorsen et al., 2015).

### 1.4 Comparison of therapeutic strategies/CBT and MBCT

Studies that examined which factors influence the effectiveness of exposure or acceptance strategies are rare. An experimental study of Olatunji et al. (2009) demonstrated that habituation is possible if anxiety is the most prominent emotion during exposition with response prevention (Olatunji et al., 2009). Fisher and Wells (2005b) demonstrated that meta-cognitive strategies are superior to other exposure strategies with respect to the tendency to show neutralising strategies.

The results of previous studies led to the consideration that emotional and cognitive aspects modulate the psychotherapeutic interventions, e.g., exposition or mindful-based strategies. The effects of these strategies for the clinical outcome are of relevance. Studies regarding neuronal parameter associated with various interventions in patients with OCD are rare.

### 1.5 Aims and hypotheses

The present study aims to clarify the extent to which strategies of acceptance and intensification overlap/differ in their neural substrates in patients with OCD. It is assumed that acceptance and intensification may influence the involvement of the fronto-cingulate system (see e.g., Messina et al., 2021). By contrast, neural responses in the PCC/precuneus are assumed to be decreased during acceptance strategy (see e.g., Messina et al., 2021).

In addition, it is assumed that the downregulation of neural responses of the amygdala may be more efficient via acceptance-based interventions compared to intensification-based interventions.

The second aim of the study was to assess if the neural correlates of acceptance and/or intensification exhibit significant changes depending on the stage of therapy in patients with OCD. We expected significant variations in orbitofrontal and medial frontal brain responses during symptom provoking tasks at the end of a multimodal inpatient treatment compared to the beginning of the therapy.

## 2 Methods

### 2.1 Subjects

Overall, 23 patients diagnosed with OCD (subtype: washing/contamination fear; DSM IV: 300.3; ICD10: F42.1/F42.2) were recruited from Schoen Clinic Roseneck, Prien am Chiemsee, Germany (see Table 1). The diagnostic assessment was administered by clinical psychologists or psychiatrists. The DSM-IV diagnoses of OCD were entered using the German version of the Structured Clinical Interview for DSM-IV (Wittchen et al., 1997).

**Table 1:** Sample characteristics.

	**OCD (*N =* 23)**
Age at entry, M (SD)	34.7 (10.87)
Male, *N* (%)	7 (30.4)
Female, *N* (%)	16 (69.6)
*Psychiatric diagnosis, N (%)*	
Comorbid anxiety disorder	6 (42.9)
Comorbid depression	7 (50)
Comorbid other	1 (7.1)
*Psychotropic drugs, N (%)*	
SSRI	6 (4.3)
SNRI	1 (4.3)
SSNRI	1 (4.3)
Mirtazapin	1 (4.3)
Bupropion	1 (4.3)
Quetiapine	5 (21.5)
Lorazepam	3 (12.9)
*Outpatient treatment, N (%)*	
Yes	20 (87)
No	2 (8.7)
No statement	1 (4.3)

M, mean; SD, standard deviation.

Written informed consent was obtained from all participants after procedures had been fully explained to them, according to the guidelines of the ethics committees of the Ludwig-Maximilians-University of Munich, Germany. The patients participated in a psychotherapeutic inpatient therapy at Schoen Clinic Roseneck, Prien am Chiemsee, Germany.

### 2.2 FMRI sessions

Patients participated in an fMRI session at the beginning of their inpatient treatment (T1), and near the end of the inpatient treatment (T2), respectively. Two patients did not participate in the fMRI session T1 and were excluded from any further fMRI analysis. The MRI data of one patient were excluded due to technical problems at T2. In addition, five patients did not complete the MRI sessions at T2 and the MRI data of one further patient had to be excluded because of motion artefacts. On this account, the data of 21 patients were included in the analysis at T1 and 14 patients in the group comparison of the comparison of T1 and T2 (see Tables 2, 3).

**Table 2:** Number of datasets included in the MRI and behavioural data (questionnaires) analysis at T1 and T2.

**Patients**	**FMRI-T1**	**FMRI-T2**	**Q-T1**	**Q-T2**
OCD	21	14	22	17

Q, questionnaires.

**Table 3:** Duration between MRI measurements (T1 and T2; days).

**Mean**	**SD**	**Median**	**Min**	**Max**
52.4	17.14	48	21	95

SD, standard deviation; min, minimum; max, maximum.

### 2.3 Questionnaires

At T1 and T2 the following questionnaires were answered by the patients to assess the therapy outcome: demographics, Obsessive-Compulsive Inventory-Revised (OCI-R; Gonner et al., 2008), Yale-Brown Obsessive Compulsive Scale (Y-BOCS; Hand and Büttner-Westphal, 1991), Beck Depression Inventory-Second Edition (BDI-II; Kühner et al., 2007), and the Acceptance and Action Questionnaire–II (AAQ-II; Hoyer and Gloster, 2013).

### 2.4 Treatment

All patients participated in intensive, multimodal inpatient treatment programme at the Schoen Clinic Roseneck, Prien am Chiemsee, Germany including both individual (50 min/1–2 times a week), group (psycho)therapy (occupational therapy, sports therapy, disorder-specific group). In addition, psychopharmacological treatment was offered to patients with the respective therapeutic indication. Diverse cognitive-behavioural elements were included in the therapy, like psychoeducation about OCD, individualised case formulation, confrontation/in vivo ERP, modification of obsessive thought/beliefs. All therapies were conducted by clinical psychologists and/or psychiatrists trained in CBT; therapies were supervised by experienced psychotherapists. The inpatient stay lasted between 2–3 months and included 50–350 min of exposure therapy depending on the individual.

### 2.5 Paradigm

**Figure 1:**
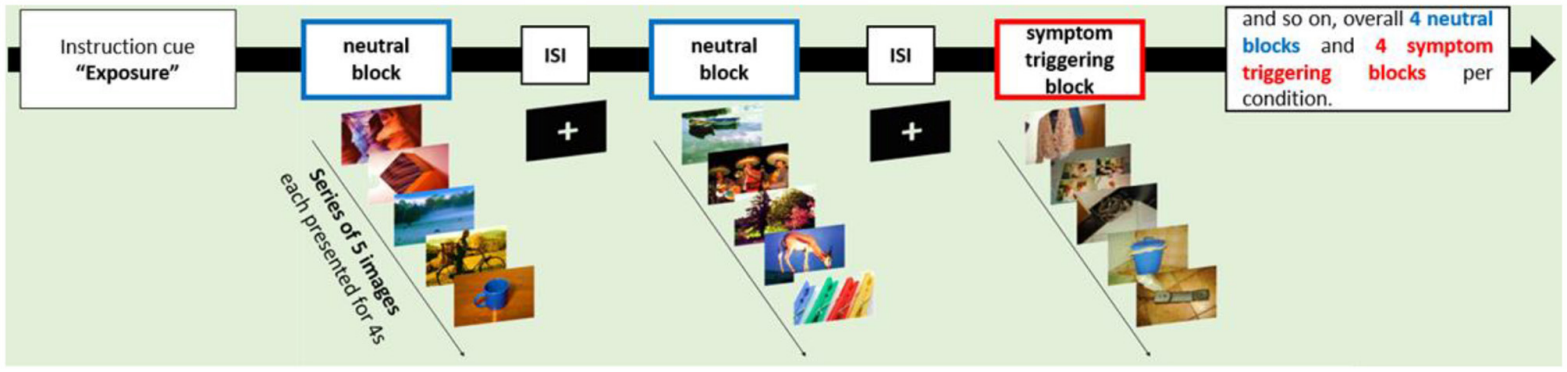
Paradigm. ISI, interstimulus interval.

Functional MRI data was acquired during a symptom provocation task in which participants saw neutral images or symptom triggering visual cues in a blocked design (see Figure 1). Symptom triggering cues consisted of images taken by participants themselves, to ensure that the individual OCD symptomatology was properly targeted (see also Schiepek et al., 2013). At least 8 different image motifs that trigger strong unpleasant reactions should be selected for the image set (*N* = 32, image format 1,280 × 960). The pictures showed specific triggers for compulsive behaviour and/or obsessive thoughts, such as objects that are dirty or often touched like door handles or toilets. Neutral pictures were taken from the “International Affective Picture System” (IAPS) (Lang et al., 2008).

A total of 32 individual symptom-triggering images and 32 neutral images were presented in the study. All triggering pictures were presented to patients before the first MRI session in order to reduce primacy, surprise, and habituation effects. In addition, patients were asked to rate the pictures using a scale from 0 (no reaction) to 4 (strong reaction; total mean score = 2.94). During the MRI session, each image was presented twice in a pseudo-randomised order for 4 seconds each. The same pictures were used for the first and second MRI session. Pictures were presented using Presentation^®^ (Neurobehavioral Systems; https://www.neurobs.com). After the MRI scan, patients were asked whether viewing the images produced comparable emotions than getting in contact with these objects in real life (scale: 0–3; 0 = not at all, 3 very much; total mean score = 1.6).

During the MRI session prior to each block with OCD triggering pictures a cue was displayed requesting participants to utilise either the acceptance strategy (ACS) or the intensification strategy (INS). The two strategies were trained by patients prior to testing by listing to verbal instructions recorded by an experimenter.

#### 2.5.1 Acceptance strategy

The aim of ACS is to accept upcoming emotions irrespective of their intensity and quality. The instruction was written based on the manual of “Training of emotional skills/competencies” (Berking, 2017).

“*I accept all of my current sensations, feelings, and thoughts. I am able to endure my condition. There is no need to change or control my feelings and thoughts. It's okay to react emotionally like I do right now*.”

#### 2.5.2 Intensification strategy

During INS, participants were instructed to expose oneself to their emotions/perceived distress and do not try to avoid them by interior distraction. The instruction was written based on the manual for OCD therapy (Lakatos and Reinecker, 1999).

“*I stay completely focused on the situation and concentrate on my feelings and physical sensations. I monitor the change of my breathing and heartbeat. I perceive and experience my unpleasant sensations as intensely as possible*.”

### 2.6 MRI acquisition and analysis

Functional MRI sessions were carried out at the Department of Radiology, University Hospital LMU, Munich, Germany. The measurements took place in a 3-Tesla MRI scanner (Ingenia 3T, Philips Healthcare, Best, The Netherlands) using a 32ch phased array head coil. A high-resolution T1-weighted MPRANGE sequence was acquired for anatomical referencing. Afterwards, functional images were obtained by a gradient echo-planar imaging sequence (3 Tesla MRI: repetition time: 2500 ms; echo time: 30 ms; FOV: 224 × 237 mm; 3 mm isotropic voxel size; 49 transverse slices; no gap).

The processing and statistical data analysis of the fMRI data were done using the BrainVoyager Software Package V21.5 (Brain Innovation, Maastricht, The Netherlands). Due to relaxation effects, the first two images were excluded from any further analysis. The pre-processing of the fMRI data included high-pass filtering (cut-off: three cycles in time course) to low frequency signal drift inherent in echo planar imaging, a slice scan time correction, spatial smoothing (Gaussian philtre with FWHM 4.0 mm), and a 3D-motion correction. Furthermore, the functional images were transferred to a standard Talairach brain. Significant BOLD activity was determined by a cross correlation of MR image pixel intensity with an expected hemodynamic response function. Voxel-wise *t*-tests were used to identify those brain areas where the signal change was significantly different between OCD related responses compared to neutral stimuli. The fMRI data of T1 and T2 were calculated in the same general linear model (GLM). For each participant the conditions “ACS”, and “INS” for T1 and T2 were calculated as regressors. To compare brain activity during the respective condition a fixed-effects-analysis was calculated [p(Bonf) < 0.050]. The following comparisons were calculated: (1) T1: ACS (OCD-related pictures vs. neutral pictures), (2) T1: INS (OCD-related pictures vs. neutral pictures), (3) T1: ACS vs. INS (OCD-related pictures vs. neutral pictures), (4) T1 vs. T2: ACS (OCD-related pictures vs. neutral pictures), (5) T1 vs. T2: INS (OCD-related pictures vs. neutral pictures), and (6) T2: ACS vs. INS (OCD-related pictures vs. neutral pictures).

### 2.7 Statistical analysis

Statistical analyses of the questionnaires were performed using SPSS version 26 with a level of significance *p* < 0.05. For the calculation of the comparison pre- and post-therapy (T1 vs. T2) a general linear model (GLM) with a repeated measurement of two levels (T1, T2) was calculated. With two stages, sphericity is a given prerequisite, so that further testing could be omitted. A chi^2^-test was performed due to the distribution of male and female in our sample and tested with a non-parametric Mann–Whitney U test if there is a gender bias in the questionnaires. This procedure was chosen because the sample was too small for a covariate to be included in the model.

## 3 Results

### 3.1 Statistical analysis

There was no significant result in the chi^2^-test or in the Mann-Whitney U test.

#### 3.1.1 Clinical results: changes in symptom severity

The comparison of Y-BOCS total score and its three subscores including subscore obsessions, subscore compulsions and subscore general revealed a significant reduction between pre-post measurements [measurements before/after intervention; Y-BOCS total F_(1, 14)_ = 32.01, *p* < 0.01, Y-BOCS subscore obsessions F_(1, 14)_ = 21.06, *p* < 0.01, Y-BOCS subscore compulsions F_(1, 15)_ = 11.847, *p* < 0.01, and Y-BOCS subscore general F_(1, 16)_ = 42.80, *p* < 0.01] (see Table 4).

**Table 4:** Psychometric data between of patients with OCD - T1 vs. T2.

**Questionnaire**	**OCD**				***p*-value**
	**T1**		**T2**		
	**M**	**SD**	**M**	**SD**	
Y-BOCS total	27.0	7.03	15.6	8.30	< 0.01^*^
Y-BOCS obsessions	14.40	4.21	8.33	3.99	< 0.01^*^
Y-BOCS compulsions	11.81	4.51	7.44	4.46	< 0.01^*^
Y-BOCS general	5.47	2.15	1.35	1.50	< 0.01^*^
OCI checking	7.53	4.11	3.29	3.29	< 0.01^*^
OCI washing	6.24	5.54	2.24	3.17	< 0.01^*^
OCI obsessing	7.59	3.83	4.06	2.68	< 0.01^*^
OCI ordering	6.63	3.96	2.94	3.02	< 0.01^*^
OCI hoarding	3.12	3.67	1.35	1.97	0.01^*^
OCI neutralising	4.29	4.03	2.47	2.85	0.02^*^
FAH	31.12	8.19	22.82	9.61	< 0.01^*^
BDI	32.00	12.99	13.19	11.40	< 0.01^*^

^*^: Significant.

The comparison of OCI total score also revealed a significant reduction between pre-post measurements [measurement before/after intervention; F_(1, 15)_ = 19.52, *p* < 0.01]. All six OCI subscores also revealed a significant reduction between pre-post measurements [measurement before/after intervention for (I) OCI checking; F_(1, 16)_ = 23.56, *p* < 0.01, (II) OCI washing; F_(1, 16)_ = 13.27, *p* < 0.01, (III) OCI obsessing; F_(1, 16)_ = 19.90, *p* < 0.01, IV) OCI ordering; F_(1, 15)_ = 12.78, *p* < 0.01, (V) OCI hoarding; F_(1, 16)_ = 26.47, *p* = 0.01, and (VI) OCI neutralising; F_(1, 16)_ = 6,18, *p* = 0.02].

Likewise, the FAH total score displays a significant reduction between pre-post measurements [measurements before/after intervention; F_(1, 16)_ = 13.07, *p* < 0.01].

Furthermore, the BDI total score also demonstrates a significant reduction between pre-post measurements [measurement before/after intervention; F_(1, 14)_ = 31.80, *p* < 0.01].

#### 3.1.2 Evaluation of OCD-relevant pictures

Finally, the evaluation of OCD-relevant pictures partly illustrated significant results in the difference between pre-post measures, namely a reduction in “amplifying emotions and thoughts through intensification strategy” [measurements before/after intervention; F_(1, 13)_ = 5.03, *p* = 0.04], an increase in “success of acceptance strategy” [measurements before/after intervention; F_(1, 15)_ = 13.36, *p* < 0.01], an increase in “acceptance strategy promotes control over sensation” [measurements before/after intervention; F_(1, 15)_ = 6.36, p =0.02], an increase in “acceptance strategy helpful” [measurements before/after intervention F_(1, 13)_ = 10.85, *p* < 0.01], and an increase in “acceptance strategy relief of unpleasant sensations” [measurements before/after intervention F_(1, 13)_ = 8.51, *p* = 0.01].

### 3.2 Neuronal responses

#### 3.2.1 Neuronal responses during ACS at T1 (N = 21)

**Figure 2:**
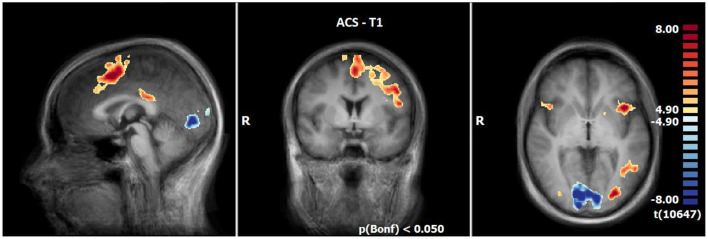
Neuronal responses during acceptance (ACS) at T1 (OCD-related pictures vs. neutral pictures); [*p*(Bonf) < 0.05, T-score: 4.9–8.0; x = 0; y = 0; z = 0].

At T1, results demonstrated enhanced OCD-associated responses in the middle frontal gyrus, the medial frontal gyrus, the inferior frontal gyrus (IFG), the left anterior insula, the superior parietal lobule, the posterior cingulate (PCC), the precuneus, the middle occipital gyrus and declive/culmen. By contrast, responses in the lingual gyrus, the superior/middle temporal gyrus and the insula right were decreased compared to the neutral condition during ACS (see Table 5, Figure 2).

**Table 5:** Neuronal responses during acceptance condition (ACS) at T1 [OCD-related pictures vs. neutral pictures, fixed effects analysis, *p*(Bonf) < 0.05, T-score: 4.9–8.0, cluster threshold: 4 voxels].

**T1: Acceptance (ACS)**								
**ACS** > **neutral**								
			**Centre of gravity**			**Size**	**T-score**	
**Brain region**	**Side**	**BA**	**X**	**Y**	**Z**	**N**	**Ø**	**Max**
Inferior frontal gyrus	R	47	48	16	3	656	5.47	6.52
Middle frontal gyrus	L	6	−29	−3	50	5,764	5.71	8.60
	L	9	−38	16	27	8,266	5.95	9.40
Medial frontal gyrus	L	6	−2	4	55	7,643	5.95	9.37
Superior frontal gyrus	L	6	−12	20	55	191	5.41	6.75
Putamen	L		−19	8	6	239	5.22	6.09
Insula	L	13	−37	18	6	4,554	5.67	9.31
Cingulate gyrus	L	23	−1	−30	26	993	5.71	7.57
Posterior cingulate	L	30	−11	−57	12	1,320	6.23	9.68
Superior parietal lobule	L	7	−27	−61	42	12046	6.68	10.78
Postcentral gyrus	L	40	−55	−25	21	240	5.41	6.38
Precuneus	R	7	21	−67	43	4,114	5.75	8.71
Middle occipital gyrus	R	19	31	−80	9	1,117	5.66	7.87
	L	19	−29	−76	18	5,911	6.14	10.19
Declive	R		38	−77	−18	939	5.43	7.03
	L		−36	−54	−11	7,644	5.98	8.94
Culmen	R		32	−60	−24	252	5.22	6.20
	L		−33	−54	−28	2,011	5.52	7.88
**ACS**<**neutral**								
			**Centre of gravity**			**Size**	**T-score**	
**Brain region**	**Side**	**BA**	**X**	**Y**	**Z**	**N**	**Ø**	**Max**
Superior temporal gyrus	R	22	53	−10	9	199	−5.19	−5.86
	R	38	52	−55	27	529	−5.43	−6.84
Middle temporal gyrus	R	21	50	−6	−11	501	−5.52	−6.89
Insula	R	13	35	−20	15	619	−5.35	−6.22
Lingual gyrus	R/L	18	3	−83	0	13,130	−6.82	−13.04

BA, Brodmann area; side, hemisphere; L, left; R, right; max, maximal t-score; Ø, average t-score; size, cluster size; voxels, number of activated voxels; x, Talairach coordinate x-axis; y, Talairach coordinate y-axis; z, Talairach coordinate z-axis; N, number of activated voxels; cluster threshold = 4 voxels.

#### 3.2.2 Neuronal responses during INS at T1 (N = 21)

**Figure 3:**
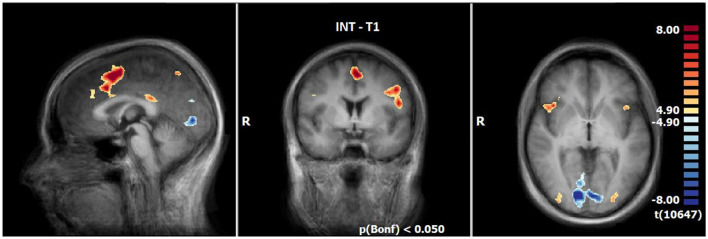
Neuronal responses during intensification (INS) at T1 (OCD-related pictures vs. neutral pictures); [*p*(Bonf) < 0.05, T-score: 4.9–8.0; x = 0; y = 0; z = 0].

INS revealed enhanced responses compared to the neutral condition especially lateral and medial frontally, in the insula (R > L), in the inferior parietal lobule, precuneus, posterior cingulate, the middle occipital gyrus and decline at T1. By contrast, the responses are decreased in the lingual gyrus, cuneus, angular gyrus and a small part of the middle frontal gyrus (see Table 6, Figure 3).

**Table 6:** Neuronal responses during intensification (INS) at T1 (OCD-related pictures vs. neutral pictures, fixed effects analysis, *p*(Bonf) < 0.05, T-score: 4.9–8.0, cluster threshold: 4 voxels).

**T1: Intensification (INS)**								
**INS** > **neutral**								
			**Centre of gravity**			**Size**	**T-score**	
**Brain region**	**Side**	**BA**	**X**	**Y**	**Z**	**N**	**Ø**	**Max**
Inferior frontal gyrus	R	9	50	5	23	566	6.29	11.10
	L	47	−29	25	−22	329	5.35	6.50
	L	9	−45	3	27	3,411	5.95	9.71
Middle frontal gyrus	R	9	44	19	25	274	5.37	6.70
	L	6	−26	−6	50	370	5.30	6.24
	L	9	−38	28	26	3,208	6.26	9.50
Medial frontal gyrus	L	32	−1	9	45	5,016	6.29	11.10
Cingulate gyrus	L	32	0	29	30	143	5.18	5.98
Posterior cingulate	R	30	13	−56	16	132	5.43	7.00
	L	23	−1	−35	24	390	5.47	7.19
	L	29	−9	−53	10	1,326	5.85	9.84
Inferior parietal lobule	L	40	−35	−52	42	7,991	7.04	13.86
Precuneus	L	19	−30	−74	30	5,168	6.35	12.15
	L	7	21	−68	42	3,949	6.01	9.05
Insula	R	13	37	15	9	2,655	6.50	11.26
	L	13	−39	17	5	520	5.36	6.64
Fusiform gyrus	L	37	−28	−41	−10	519	5.53	6.98
Middle occipital gyrus	R	19	30	−81	13	1,551	6.43	9.60
		18	32	−84	0	279	5.45	7.20
Declive	R		42	−65	−17	1,854	5.53	7.17
	L		−41	−59	−14	4,429	6.00	9.71
Culmen	R		29	−60	−26	863	5.53	7.25
			27	−42	−11	682	5.57	7.44
Pyramis	R		10	−70	−26	251	5.57	7.20
	L		−13	−71	−26	281	5.64	8.01
**INS**<**neutral**								
			**Centre of gravity**			**Size**	**t-score**	
**Brain region**	**Side**	**BA**	**X**	**Y**	**Y**	**N**	**Ø**	**Max**
Angular gyrus	R	39	47	−57	35	200	−5.51	−6.82
Middle frontal gyrus	R	10	29	63	8	184	−5.98	−7.85
Lingual gyrus/cuneus	R/L	1,718	5	−81	3	7,420	−6.25	−12.75
Cuneus	L	18	−10	−85	19	273	−5.23	−6.11

BA, Brodmann area; side, hemisphere; L, left; R, right; max, maximal t-score; Ø, average t-score; size, cluster size; voxels, number of activated voxels; x, Talairach coordinate x-axis; y, Talairach coordinate y-axis; z, Talairach coordinate z-axis; N, number of activated voxels; cluster threshold = 4 voxels.

#### 3.2.3 Comparison of neuronal responses of ACS vs. INS at T1 (N = 21)

The BOLD responses between ACS and INS (OCD-related picture vs. neutral pictures) at T1 did not differ significantly (OCD-related pictures vs. neutral pictures, fixed effects analysis, *p*(Bonf) < 0.05, T-score: 4.9–8.0, cluster threshold: 7 voxels).

#### 3.2.4 Comparison of neuronal responses of T1 vs. T2 during ACS (N = 14)

**Figure 4:**
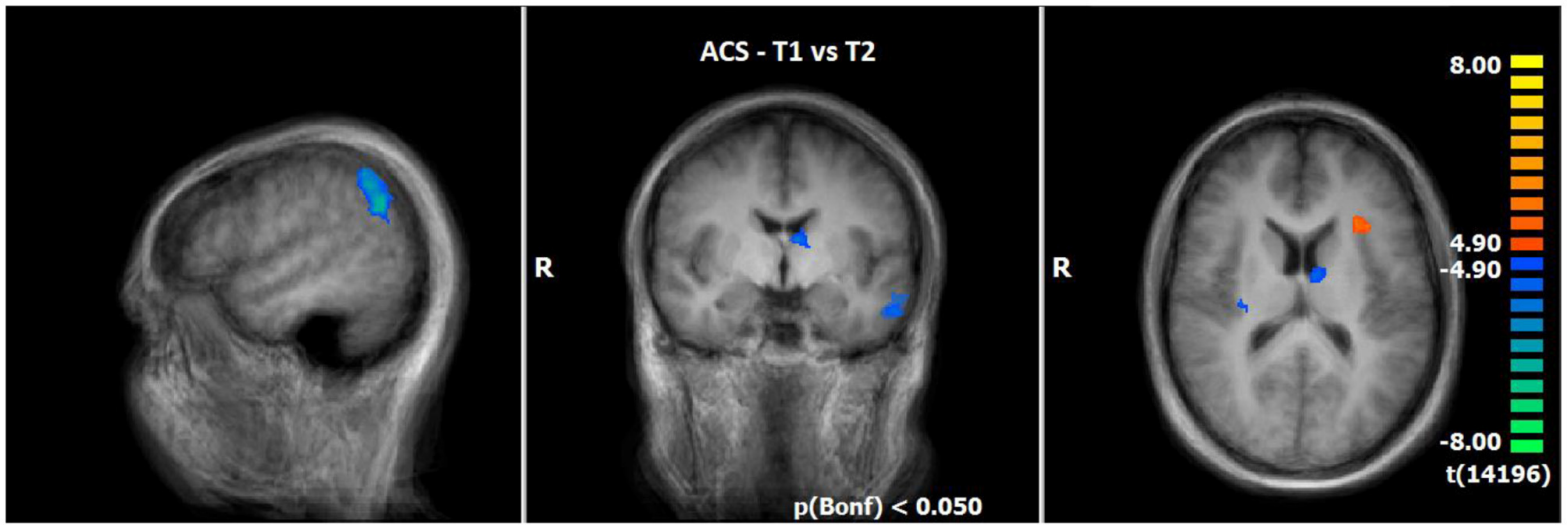
Comparison of neuronal responses during ACS between T1 and T2 (OCD-related pictures vs. neutral pictures); [*p*(Bonf) < 0.050, T-score: 4.9–8.0; x = 50; y = −5; z = 15].

The comparison of BOLD responses during ACS before and after therapeutic interventions demonstrated an increased neuronal activity especially in the frontal cortex (including the medial frontal gyrus/middle frontal gyrus/inferior frontal gyrus), parietal areas (e.g., inferior parietal lobule/angular gyrus/supramarginal gyrus), and subcortical areas (e.g. thalamus/anterior nucleus, lentiform gyrus/putamen/claustrum) in T2 as compared to T1. By contrast, neuronal responses were smaller in the left insular cortex (see Table 7; Figure 4).

**Table 7:** Comparison of neuronal responses during ACS between T1 and T2 [OCD-related pictures vs. neutral pictures, fixed effects analysis, *p*(Bonf) < 0.050, T-score: 4.9–8.0, cluster threshold: 4 voxels].

**Acceptance strategy (ACS): T1 vs. T2**								
			**Centre of gravity**			**Size**	**T-score**	
**Brain region**	**Side**	**BA**	**X**	**Y**	**Z**	**N**	**Ø**	**Max**
**T2** > **T1**								
Medial frontal gyrus	R	6/8	3	36	37	245	5.12	5.74
	R	8	21	21	41	157	5.20	5.80
Inferior frontal gyrus	L	47	−37	31	−8	208	5.26	6.25
Inferior temporal gyrus	L	21/20	−56	−11	−18	1,110	5.39	6.66
Thalamus/caudate body	L		−8	−4	14	355	5.24	6.20
Lentiform nucleus/putamen/claustrum	R		30	−19	12	221	5.20	6.13
Inferior parietal lobule/angular gyrus/supramarginal gyrus	R	40	49	−55	38	4,302	5.75	7.68
Cuneus	R		25	−84	30	150	5.25	5.86
**T1** > **T2**								
Insula	L	13	−31	21	14	386	5.31	6.76
	L		−35	12	1	110	5.25	6.20

BA, Brodman area; side, hemisphere; L, left; R, right; max, maximal t-score; Ø, average t-score; size, cluster size; voxels, number of activated voxels; x, Talairach coordinate x-axis; y, Talairach coordinate y-axis; z, Talairach coordinate z-axis N, number of activated voxels; cluster threshold = 4 voxels.

#### 3.2.5 Comparison of neuronal responses of T1 vs. T2 during INS (N = 14)

**Figure 5:**
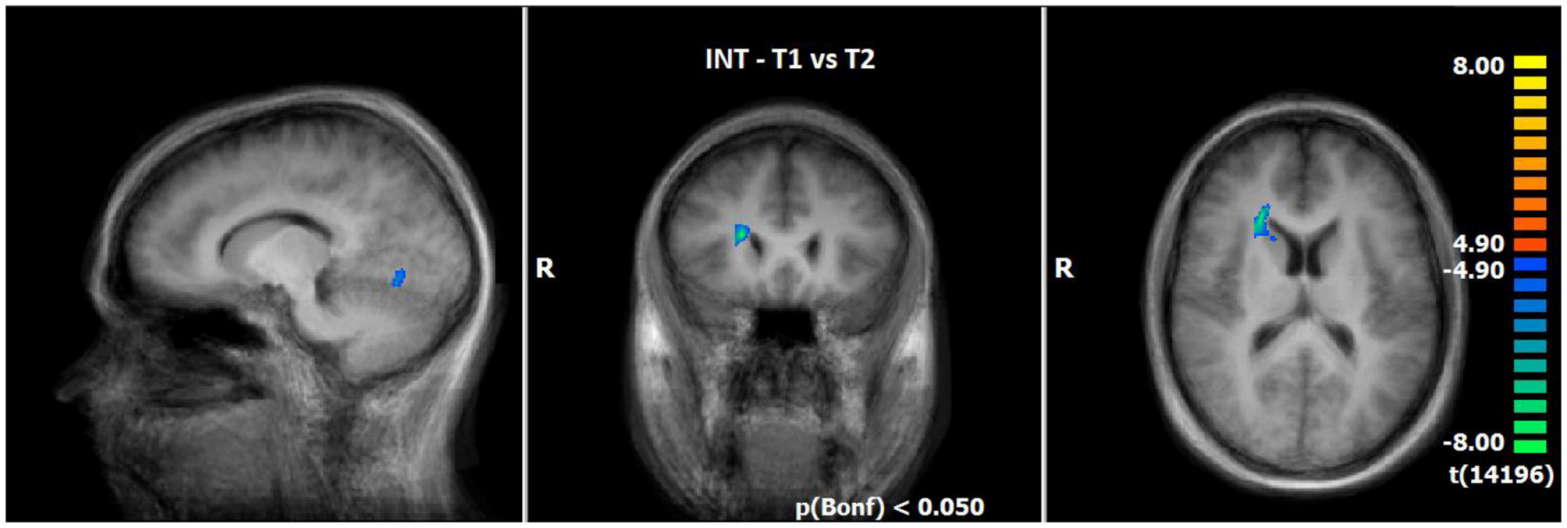
Comparison of neuronal responses during INS between T1 and T2 (OCD-related pictures vs. neutral pictures); [*p*(Bonf) < 0.05, T-score: 4.9–8.0; x = 12; y = 22; z = 15].

Utilising the INS, a slightly enhanced neuronal response has been shown in the cerebellum/culmen as well as caudate body at T2 as compared to T1 (see Table 8; Figure 5).

**Table 8:** Comparison of neuronal responses during INS between T1 and T2 [OCD-related pictures vs. neutral pictures, fixed effects analysis, *p*(Bonf) < 0.05, T-score: 4.9–8.0, cluster threshold: 4 voxels].

**Intensification (INS): T1 vs. T2**								
			**Centre of gravity**			**Size**	**T-score**	
**Brain region**	**Side**	**BA**	**X**	**Y**	**Z**	**N**	**Ø**	**Max**
**T2** > **T1**								
Lingual gyrus	R	18/19	12	−65	−5	115	5.30	6.36
Caudate body	R		12	22	15	887	6.03	9.72

BA, Brodman area; side, hemisphere; L, left; R, right; max, maximal t-score; Ø, average t-score; size, cluster size; voxels, number of activated voxels; x, Talairach coordinate x-axis; y, Talairach coordinate y-axis; z, Talairach coordinate z-axis; cluster threshold, 4 voxels.

#### 3.2.6 Comparison of neuronal responses of ACS vs. INS in T2 (N = 14)

**Figure 6:**
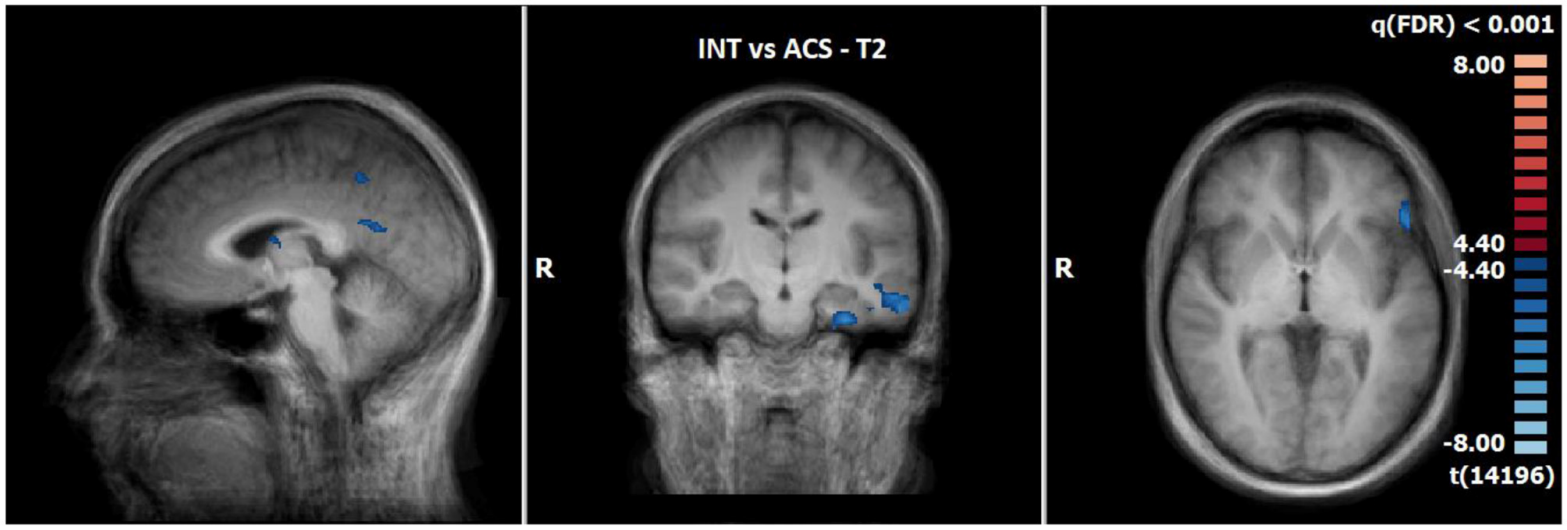
Comparison of neuronal responses during T2 between ACS and INS [OCD-related pictures vs. neutral pictures, *p*(FDR) < 0.001, T-score: 4.4–8.0; x = −3; y = −15; z = 1].

The comparison of BOLD responses at the end of therapy revealed more pronounced reactions during ACS as compared to INS particularly in the precentral gyrus/inferior frontal gyrus, the postcentral gyrus, the inferior/middle temporal gyrus and limbic areas including the parahippocampal gyrus (see Table 9; Figure 6).

**Table 9:** Comparison of neuronal responses during T2 between ACS and INS [OCD-related pictures vs. neutral pictures, fixed effects analysis, *p*(FDR) < 0.001, cluster threshold: 7 voxels, T-score: 4.4–8.0].

**T2: ACS** > **INS**								
			**Centre of gravity**			**Size**	**T-score**	
**Brain region**	**Side**	**BA**	**X**	**Y**	**Z**	**N**	**Ø**	**Max**
Inferior frontal gyrus	L	47	−34	33	−9	208	4.72	5.63
			−49	26	1	245	4.41	4.91
		9	−41	5	26	1,269	4.96	6.64
Precentral gyrus	L	9	−35	13	40	195	4.89	6.07
Postcentral gyrus	L	3	−34	−30	52	473	5.04	6.53
			−43	−20	44	300	4.80	6.39
Precuneus	L	7	6	48	43	205	4.77	5.80
Posterior cingulate gyrus	L	23	−1	−52	21	194	4.72	5.55
Middle temporal gyrus	L	21	−51	−18	−16	1,118	4.97	6.93
Superior temporal gyrus	L	38	−31	3	−28	457	4.81	5.84
Caudate body	L	–	−8	−4	14	465	4.72	5.75
Uncus	L	20	−30	−16	−26	503	5.18	7.19

BA, Brodman area; side, hemisphere; L, left; R, right; max, maximal t-score; Ø, average t-score; size, cluster size; voxels, number of activated voxels; x, Talairach coordinate x-axis; y, Talairach coordinate y-axis; z, Talairach coordinate z-axis; cluster threshold, 7 voxels.

## 4 Discussion

To the best of our knowledge, this study is the first to compare the neurobiological basis of emotion processing in patients with OCD during acceptance strategy (ACS) vs. intensification strategy (INS) after a psychotherapy.

### 4.1 Clinical outcome of psychometric data

The inpatient multimodal therapy programme was shown to be successful as it resulted in a significant reduction in all the symptom scales. Obsessive thoughts and compulsive behaviour decreased as well as depressive symptoms. In addition, the patients were able to gain cognitive flexibility. During the therapy, the patients learned to use INS and ACS better, whereby ACS was rated as more suitable for them regarding emotion regulation.

### 4.2 Neuronal responses before treatment

Results of ACS at T1 are not in line with findings of the recent meta-analysis of Messina et al. (2021), indicating a specific deactivation of the PCC when the acceptance strategy was compared to a control condition (e.g., react naturally or focus on emotions). However, the meta-analysis did not include patients with OCD, but examined healthy subjects and patients with anxiety disorder. In the current study no functional differences between ACS and INS were found. This result may indicate that at the beginning of therapy both strategies were equally helpful and/or not yet understood or applicable to the same extent as they were at the end of therapy. In line with other studies (Schiepek et al., 2013), typical brain regions for OCD showed a stronger activation during the presentation of OCD relevant information as compared to neutral pictures both in ACS and INS. These brain regions are important for processing emotional (e.g., left anterior insula), for the modulation of attention (e.g., precuneus), and for executive functions (e.g., inferior/medial/middle frontal gyrus, DLPFC, cingulate gyrus).

The enhanced responses in superior parietal areas [e.g., supplementary motor area (SMA) and putamen] may be associated to motor information processing, (motor-)learning, and control of behavioural programs (Aouizerate et al., 2004; Ell et al., 2006, 2011; Marchand et al., 2008). They are in accordance with other studies (Schiepek et al., 2013; Yu et al., 2022). The stronger activation of occipital regions for neutral pictures in ACS and INS is also in line with previous studies and seems to be a neuronal correlate of a less emotional involvement and stronger visual or “neutral” processing (Maywald et al., 2022a,b).

## 5 Limitations

The following limitations must be considered in this study: first, the small sample size is limiting the statistical power. However, the results of the present study are in line with those of former studies in patients with OCD. Second, due to the small sample size a differentiation regarding gender and handedness was not realisable in the MRI analysis. Concerning the results of the questionnaires, statistical analysis did not show any gender-related effect. Third, it would have been helpful to include further control conditions (e.g., natural control condition, see cf. Messina et al., 2021; Kolar et al., 2023) to further elucidate underlying neurobiological effects especially at the beginning of the therapy.

The imaging paradigm would have benefitted from the inclusion of further control conditions to further disentangle functional differences that might has been present in the sample before the beginning of the therapy. In addition, it could have been useful to include a larger sample of different pictures of each category to further reduce habituation effects.

### 5.1 Functional variations after treatment

Utilising the acceptance strategy, there was an increase in activity in the left inferior frontal gyrus [BA47] at T2 compared to T1, a brain region that is involved in emotion processing and has been associated with emotion regulation disorders (Lueken et al., 2013; Maywald et al., 2022a; Yu et al., 2022). Goncalves et al. (2015) explored obsessive-compulsive disorder (OCD)-related abnormalities in white matter connectivity for a core region associated with inhibitory control, the inferior frontal gyrus (IFG). Their results suggest significant alterations in structural connectivity, probably associated with myelination and axonal abnormalities in the IFG of OCD patients (Goncalves et al., 2015). Lueken et al. (2013) found an increase in IFG activity in patients with panic disorder and interpreted their result as an increased inhibitory response to a threatening stimulus indicating modified top-down processing. Patients with OCD showed a decrease in inhibitory control in different studies (Penades et al., 2007; Gillan et al., 2011; Norman et al., 2019). Yu et al. (2022) found a decrease in left IFG activity in their meta-analysis compared to healthy controls. We propose that an increase of neuronal activity in the left IFG is a neural correlate of improved top-down inhibitory control in OCD patients after therapy using ACS.

In contrast to Messina et al. (2021) this study found that ACS involves more executive brain structures compared to the control condition. This specific observation may be due to the OCD patient sample and may be considered a therapy effect. We suppose that these areas are involved in “Exposure and Response Prevention” and increases in patients with OCD with better inhibitory control. Furthermore, all increased neural activity occurred only in the left hemisphere (contrast INS vs. ACS at T2). There is evidence that mediation may cause increased neural responses especially in the left IFG, and enhance left frontal cerebral blood flow lateralization (Tang et al., 2015; Izzetoglu et al., 2020). Moreover, meditation can lead to improved performances and improved inhibitory control in a STROOP-test (Izzetoglu et al., 2020). We hypothesise that the acceptance strategy leads to a stronger left hemispheric lateralisation in patients with OCD, especially in frontal areas like the IFG [BA47, BA9].

The left posterior cingulate gyrus/cuneus seems to play a role in down-regulation emotion e.g., with acceptance strategy, emotional avoidance of words, self-distraction, or reappraisal strategies (Kanske et al., 2010; Koenigsberg et al., 2010; Benelli et al., 2012; Messina et al., 2021), and an increase in PCC/precuneus activity was observed after psychotherapy (Buchheim et al., 2013; Maywald, 2023). It is therefore hypothesised that PCC activity increased while using the ACS strategy in the context of progressed psychotherapy.

In accordance with other studies (Schiepek et al., 2013; Norman et al., 2019) we found a decrease in the left anterior insula. Functional neuroimaging studies have linked activations of the insular cortex to various aspects including subjective disgust related information/emotional perception of emotional states (Craig et al., 2002), aversive valence of information (Anderson and Savage, 2004) and emotional tasks (Phan et al., 2002). In addition, insula plays an important role in a wide range of psychiatric disorders including obsessive-compulsive disorder (Avery et al., 2014; Buyukturkoglu et al., 2015; Maywald et al., 2022b). A decreased neuronal response at T2 compared to T1 may indicate decreased emotional responses during the processing of OCD-relevant information.

The caudate nucleus as well as the putamen, the supramarginal and angular gyrus all showed an increase in neuronal activity with therapy during ACS. These brain structures are associated with the cognitive or attentional/spatial network (Saxena and Rauch, 2000; Schiepek et al., 2013). Different authors (Chen et al., 2004; Baioui et al., 2013) found an increased caudate nucleus activity when comparing patients with OCD (also washing subgroup) to a healthy control group, or an increased nucleus caudatus/putamen/SMG/angular gyrus activity before therapy (Nakatani et al., 2003; Schiepek et al., 2013). One would therefore expect that neuronal activity in these structure should be reduced after psychotherapy, but the opposite effect was determined in the current study and the study of Freyer et al. (2011). In our study both ACS and INS showed increased nucleus caudatus activity after therapy. This result may be influence by the comparison group: the current study focused on intraindividual effects whereas other studies focused on the comparison to a control group. The Research Domain Criteria (RDoC) approach suggests that even if the manifestation of symptoms are similar (e.g., excessive hand washing), the underlying processes of these symptoms may vary from person to person (Cuthbert, 2022; Kalanthroff and Wheaton, 2022). In analogy, neurobiological processes could also differ. Another explanation of this contradictory finding addresses the possibility that this structure may be involved in both the initiation of motor action and inhibition, and that certain substructures similar to the thalamus (Kumar et al., 2022) exist that need further investigations.

It is assumed that cognitive control, learning processes, and complex visual processing have an increased impact during the presentation of symptom triggering pictures at the end of therapy as compared to the beginning. This may indicate that ACS is associated with improved neuronal responses within the cognitive network at the end of therapy as compared to the beginning, and as compared to INS. This suggests a changed strategy for processing disorder-specific information at the end of a therapy. FMRI results are in accordance with the subjective questionnaire assessments of patients, in which the acceptance strategy was rated as more efficient, compared to the intensification strategy. This result is also in accordance to another experimental study with similar paradigm of Kolar et al. (2023). Brain responses during the utilisation of the intensification strategy differed between T1 and T2 in the lingual gyrus. This region was also increased in the contrast provoking vs. neutral pictures at T1. This points towards a visual and less emotional processing.

Taking these results together both strategies showed an improvement after therapy, but ACS showed enhanced cognitive processing and less emotional responses than INS, and was accepted more by patients, which could mean that ACS is the ERP strategy to favour.

## 6 Conclusion

We assume that it requires a learning process over therapy to reveal significant differences in neural activity between the ACS and the INS in our specific setting. At the end of the therapy, especially ACS is associated with improved neuronal responses within the cognitive network compared to INS. Furthermore, ACS seems to mobilise neuronal activations under therapy, especially in the left hemisphere. Both strategies showed reduced neuronal activations in the emotional network and increased activations in the visual network after the therapy. Neutral pictures in contrast to OCD pictures lead to stronger visual than emotional processing in occipital regions in ACS and INS at the beginning of the therapy. Increased left IFG activity in ACS condition was interpreted as a better inhibitory top-down process and increased PCC as a better reappraisal strategy after therapy. Overall, we assume ACS strategy is more efficient than INS as rated by the patients and as in accordance with neurobiological findings.

## Data availability statement

The original contributions presented in the study are included in the article/supplementary material, further inquiries can be directed to the corresponding author.

## Ethics statement

The studies involving humans were approved by Ethikkommission der medizinischen Ludwig-Maximilians-Universität München. The studies were conducted in accordance with the local legislation and institutional requirements. The participants provided their written informed consent to participate in this study.

## Author contributions

SK: Conceptualisation, Formal analysis, Project administration, Supervision, Writing—original draught. MM: Formal analysis, Supervision, Writing—original draught, Writing—review & editing. CS: Writing—review & editing. CH: Formal analysis, Writing—review & editing. JN: Formal analysis, Investigation, Writing—review & editing. DK: Investigation, Writing—review & editing. SG: Writing—review & editing. NT: Writing—review & editing. OP: Conceptualisation, Writing—review & editing. MP: Conceptualisation, Investigation, Project administration, Supervision, Writing—original draught. UV: Conceptualisation, Funding acquisition, Project administration, Supervision, Writing—original draught.
